# Comparison of feature selection and classification for MALDI-MS data

**DOI:** 10.1186/1471-2164-10-S1-S3

**Published:** 2009-07-07

**Authors:** Qingzhong Liu, Andrew H Sung, Mengyu Qiao, Zhongxue Chen, Jack Y Yang, Mary Qu Yang, Xudong Huang, Youping Deng

**Affiliations:** 1 Department of Computer Science, New Mexico Tech, Socorro, NM 87801 USA; 2 Institute for Complex Additive Systems Analysis, New Mexico Tech, Socorro, NM 87801, USA; 3 Biostatistics Epidemiology Research Design Core, Center for Clinical and Translational Sciences, The University of Texas Health Science Center at Houston, Houston, TX 77030, USA; 4 Harvard University P. O. Box 400888, Cambridge, MA 02140-0888, USA; 5 National Human Genome Research Institute, National Institutes of Health (NIH), U.S. Department of Health and Human Services, Bethesda, MD 20852, USA; 6 Conjugate and Medicinal Chemistry Laboratory, Division of Nuclear Medicine and Molecular Imaging and Center for Advanced Medical Imaging, Department of Radiology, Brigham and Women's Hospital and Harvard Medical School, Boston, MA 02115, USA; 7 SpecPro, Vicksburg, MS 39180, USA; 8 Department of Biology Science, The University of Southern Mississippi, 118 College Dr., Hattiesburg, MS 39406, USA

## Abstract

**Introduction:**

In the classification of Mass Spectrometry (MS) proteomics data, peak detection, feature selection, and learning classifiers are critical to classification accuracy. To better understand which methods are more accurate when classifying data, some publicly available peak detection algorithms for Matrix assisted Laser Desorption Ionization Mass Spectrometry (MALDI-MS) data were recently compared; however, the issue of different feature selection methods and different classification models as they relate to classification performance has not been addressed. With the application of intelligent computing, much progress has been made in the development of feature selection methods and learning classifiers for the analysis of high-throughput biological data. The main objective of this paper is to compare the methods of feature selection and different learning classifiers when applied to MALDI-MS data and to provide a subsequent reference for the analysis of MS proteomics data.

**Results:**

We compared a well-known method of feature selection, Support Vector Machine Recursive Feature Elimination (SVMRFE), and a recently developed method, Gradient based Leave-one-out Gene Selection (GLGS) that effectively performs microarray data analysis. We also compared several learning classifiers including K-Nearest Neighbor Classifier (KNNC), Naïve Bayes Classifier (NBC), Nearest Mean Scaled Classifier (NMSC), uncorrelated normal based quadratic Bayes Classifier recorded as UDC, Support Vector Machines, and a distance metric learning for Large Margin Nearest Neighbor classifier (LMNN) based on Mahanalobis distance. To compare, we conducted a comprehensive experimental study using three types of MALDI-MS data.

**Conclusion:**

Regarding feature selection, SVMRFE outperformed GLGS in classification. As for the learning classifiers, when classification models derived from the best training were compared, SVMs performed the best with respect to the expected testing accuracy. However, the distance metric learning LMNN outperformed SVMs and other classifiers on evaluating the best testing. In such cases, the optimum classification model based on LMNN is worth investigating for future study.

## Introduction

In proteome research, high-throughput mass spectrometry (MS) establishes an effective framework for biomedical diagnosis and protein identification [1]. A mass spectrum data sample includes a sequence of mass/charge (m/z) ratios. Two types of mechanisms, low resolution and high resolution, that typically contain more than 10,000 data points ranging from 500 Da to 20000 Da, are used in mass spectrometry.

Mass spectrum data mining usually contains four steps: preprocessing, feature extraction or peak detection, feature selection and classification. Sometimes preprocessing and peak detection are merged as preprocessing. The main task in preprocessing is to purify the data and systematically represent the data for the following steps. The MS data contain two kinds of noise that damage the classification result: electric noise and chemical noise. MS data is generated with chemical noise through matrix or ion overloading, and the noise usually shows up as a baseline along the spectrum. Baseline correction computes the local minimum value, draws a baseline represented as the background noise, and subtracts the baseline from the spectrum. Williams *et al *[2] proposed a robust algorithm for computing the baseline correction of MALDI-MS spectra. Alternatively, because electronic noise is generated from the electronic instrument and is usually randomly distributed in the spectra, Chen *et al *[3] designed a wavelet-based de-noising that applies wavelet transformation and removes a certain amount of lower value wavelet coefficients. The de-noised data are normalized to systematically represent the spectra. The next crucial step is to extract features from the spectra and then form the initial complete feature set. The simplest way is to extract every data point as a discriminative feature and generate a huge feature set including more than 15,000 features [4,5]. A more elaborate algorithm for peak detection and alignment is also available to perform an even more aggressive feature extraction [6-8].

To classify MALDI MS data, peak detection, feature selection, and classifier are generally important to obtain the final results. To compare public peak detection algorithms, Yang *et al*. [9] recently conducted an experimental study using five single spectrum based peak detection algorithms including Cromwell [10], CWT [11], PROcess [12], LMS [13], and LIMPIC [14]. That study did not compare feature selection and classifiers for MALDI-MS data. "The curse of dimensionality" in MS data requires a powerful feature selection algorithm to choose the discriminative feature subset. While distance metric learning has drawn many researchers' attention, researchers recognize that different classifiers yield different results. Therefore, a comprehensive experimental study that compares these powerful methods of feature selection and different learning classifiers for the classification of MALDI-MS data has been sorely needed.

Support Vector Machine Recursive Feature Elimination (SVMRFE) [15] is a very popular method for feature selection based on the backward feature elimination that recursively removes the least ranking feature. Originally proposed for microarray data analysis, it has been widely used for feature selection in different areas including MS data analysis [16]. Recently, Tang *et al*. designed a method of feature selection called the gradient based leave-one-out gene selection (GLGS) for classifying microarray data. The authors concluded that GLGS outperforms SVMRFE in microarray data analysis [17], a finding that our previous work corroborates in that we found that GLGS also effectively classified microarray data [18]. To reach a more definitive understanding of how methods compare, we evaluated two methods of feature selection as well as popular learning classifiers in an experimental study on MALDI-MS data.

## Methods

### Preprocessing MALDI-MS data

Mass spectrum data has high dimensionality within a small sample size. Both chemical and electrical noises are involved in the signal, and the redundancy of the spectra, different reference points, and unaligned feature points increase the computational intensity and decrease the classification accuracy. In this section, we explain the preprocessing methods, including spectra re-sampling, wavelet de-noising, baseline correction, normalization, peak detection and alignment.

#### Spectra re-sampling and wavelet de-noising

Mass spectrum data presents in a discrete format along intervals that are not equal in the whole spectrum. For high-resolution data, the high-frequency noise and redundant data points harm the quality of the dataset. So, we have to set the common low-frequent mass value to every sample spectrum to have a unified representation. By using spline interpolation, we resample the data and confine the interval to a unified size. Before re-sampling, the sample spectrum has little variation from the true spectrum. The data is re-sampled to a standard discrete data that could be analyzed in a frequency domain. The electrical noise is generated in an almost randomly distributed way during the mass spectrum acquisition by the instrument. The next step is to use discrete wavelet transform to eliminate the electrical noise. By applying a wavelet transform, the original signal is decomposed into multi-level wavelet coefficients. By setting up a threshold value, given percentiles of lower value coefficients are removed. Then, we apply a polynomial filter of a second order to smooth the signal and improve data quality.

#### Baseline correction and normalization

Chemical contamination introduces the baseline effect and changes the true protein distribution. To minimize chemical noise, the baseline is subtracted from the spectrum. To obtain the baseline, the local minima are computed by assigning a shifting window size of 30 and a step size of 30. Then, we use spline interpolation to fit the baseline. After smoothing, the baseline is subtracted from all spectra. To compare sample spectra, we need to normalize the spectra using its total ion current to represent the data in a systematic scale.

#### Peak detection and qualification

The final feature acquisition of MS data is to obtain the peak position and its magnitude. Peak is the position of maximum intensity in a local area in spectrum, and particularly in mass spectrum, it refers to the mass location where ion count is the largest in a local m/z zone. The peak is identified where the first derivative is changing from a positive to a negative. In our mass spectrum experiment, the peak detection method proposed by Coombes *et al *[19] is performed on a mean spectrum rather than individual spectra. We used the ad hoc method based on signal-to-noise ratio to select the large peaks based on the preprocessing method described in reference [6].

### Feature selection

To address the "curse of dimensionality" problem, three strategies have been proposed: filtering, wrapper and embedded methods. Filtering methods select subset features independently from the learning classifiers and do not incorporate learning. One of the weaknesses of filtering methods is that they only consider the individual feature in isolation and ignore possible interactions. Yet, the combination of these features may have a combination effect that does not necessarily follow from the individual performances of the features in that group. One of the consequences of the filtering methods is that we may end up with many highly correlated features; yet, any highly redundant information will worsen the classification and prediction performance. Furthermore, a limit on the number of features chosen may preclude the inclusion of all informative features.

To avoid the weakness in filtering methods, wrapper methods wrap around a particular learning algorithm that can assess the selected feature subsets in terms of the estimated classification errors and then build the final classifier [20]. Wrapper methods use a learning machine to measure the quality of the subsets of features. One recent well-known wrapper method for feature selection is SVMRFE proposed by Guyon *et al*. [15], which refines the optimum feature set by using the Support Vector Machine (SVM). The idea of SVMRFE is that the orientation of the separating hyper-plane found by the SVM can be used to select informative features. If the plane is orthogonal to a particular feature dimension, then that feature is informative, and vice versa. In addition to microarray classification, SVMRFE has been widely used in other high-throughput biological data analysis including a proteomics study [16] and non-bioinformatics areas involving feature selection and pattern classification situations [21]. The recursive elimination procedure of SVMRFE is listed as follows:

(1) Initial ranked feature set R = []; feature set S = [1,..., d];

(2) Repeat until all features are ranked

(a) Train a linear SVM with all the training data and variables in S;

(b) Compute the weigh vector;

(c) Compute the ranking scores for features in S;

(d) Find the feature with the smallest ranking score;

(e) Update R: R = R [e, R];

(f) Update S: S = S - [e];

(3) Output: Ranked feature list R.

Wrapper methods can noticeably reduce the number of features and significantly improve the classification accuracy [22]. However, wrapper methods have the drawback of having a high computational load. With better computational efficiency and similar performance to wrapper methods, embedded methods simultaneously process feature selection with a learning classifier. To deal with the feature selection in microarray data classification, Tang *et al*. also proposed two gene selection methods: leave-one-out calculation sequential forward selection (LOOCSFS) and GLGS that is based on the calculation of the leave-one-out cross-validation error of LS-SVM [17]. The GLGS algorithm can be categorized as an embedded method that differs greatly from previous wrapper and embedded approaches because the GLGS optimizes the evaluation criterion derived in a supervised manner in a transformed space with significantly reduced dimensions compared to the original space as it selects genes from the original gene set based on the results of the optimization. According to presented experimental results, the GLGS method is more appealing given it has the lowest generalization error [17].

Based on the above explanation, we employed SVMRFE and GLGS algorithms for feature selection in our experimental study.

### Learning classifiers

#### Support vector machines

SVM [23] has been widely used in classification. It constructs an optimal hyperplane decision function in feature space that is mapped from the original input space by using kernels, briefly introduced as follows:

Let x_*i *_denote the *i*^th ^feature vector in the original input space and z_*i *_denote the corresponding vector in the feature space, z_*i *_= Φ (x_*i*_). Kernel function *k*(x_*i*_; x_*j*_) computes the inner product of two vectors in the feature space and defines the mapping function:

(1)

Three types of commonly used kernel functions are:

Linear Kernel *k*(x_*i*_; x_*j*_) = x_*i*_•x_*j*_

Polynomical Kernel *k*(x_*i*_; x_*j*_) = (1 + x_*i*_•x_*j*_)^*p*^

Gaussian Kernel *k*(x_*i*_; x_*j*_) = exp(-||x_*i *_- x_*j*_||^2^/2*σ*^2^)

For a typical classification problem with *l *training samples (x_1_, y_1_),..., (x_*l*_, y_*l*_) where *y*_*i *_∈ {+1, -1}, finding the discriminant function *f*(*x*) = *w*•Φ (x) + *b *with the following optimization problem.

(2)

This optimization problem is usually solved in its dual form

(3)

#### Distance metric learning

Depending on the availability of training examples, the algorithms of distance metric learning can be divided into two categories: supervised distance metric learning and unsupervised distance metric learning. With the given class labels for training samples, supervised distance metric learning can be divided into global distance metric learning and local distance metric learning. The global learns the distance metric in a global sense, i.e., to satisfy all the pairwise constraints. The local approach is to learn the distance metric in a local setting, i.e., only to meet local pairwise constraints.

Unsupervised distance metric learning is also called manifold learning. Its main idea is to learn an underlying low-dimensional manifold whereby the geometric relationships between most of the observed data are preserved. Every dimension reduction approach works by essentially learning a distance metric without label information. Manifold learning algorithms can be divided into global linear dimension reduction approaches, including Principle Component Analysis (PCA) and Multiple Dimension Scaling (MDS), global nonlinear approaches, for instance, ISOMAP [24], local linear approaches, including Locally Linear Embedding (LLE) [25] and the Laplacian Eigenmap [26].

In supervised global distance metric learning, the representative work formulates distance metric learning as a constrained convex programming problem [27]. In local adaptive distance metric learning, many researchers presented approaches to learn an appropriate distance metric to improve a KNN classifier [28-32]. Inspired by the work on neighborhood component analysis [30] and metric learning with the use of energy-based models [33], Weinberger *et al*. proposed a distance metric learning for Large Margin Nearest Neighbor classification (LMNN). Specifically, the Mahanalobis distance is optimized with the goal that the k-nearest neighbors always belong to the same class while examples from different classes are separated by a large margin [34]. The LMNN has several parallels to learning in SVMs. For example, the goal of margin maximization and a convex objective function is based on the hinge loss. In multi-classification, the training time of SVMs scales at least linearly in the number of classes. By contrast, LMNN has no explicit dependence on the number of classes [34]. We introduce the idea of LMNN as follows:

Given a training set of n labeled samples and the corresponding class labels , the binary matrix *y*_*ij *_∈ {0, 1} indicates whether or not the labels *y*_*i *_and *y*_*j *_match. And *η*_*ij *_∈ {0, 1} indicates whether *x*_*j *_is a target neighbor of *x*_*i*_. Both matrices *y*_*ij *_and *η*_*ij *_are fixed during training. The goal is to learn a linear transformation L: R^*d *^→ R that optimizes KNN classification. The transform is used to compute squared distance as

(4)

The cost function is given as follows:

(5)

Where [z]_+ _= max(z,0) denotes the standard hinge loss and the constant C > 0. The first term penalizes large distances between each input and its target neighbors and the second term penalizes small distances between each input and all other inputs that do not share the same label. The optimization of eq. (5) can be reformulated as an instance of semidefinite programming (SDP) [35] and the global minimum of eq. (5) can be efficiently computed. Mahalanobis distance metric M = L^*T*^L, eq. (4) is

(6)

Slack variables *ξ*_*ij *_for all pairs of differently labeled inputs are introduced so that the hinge loss can be mimicked. The resulting SDP is given by:

Minimize

(7)

Subject to

(1) (*x*_*i *_- *x*_*l*_)M(*x*_*i *_- *x*_*l*_)-(*x*_*i *_- *x*_*j*_)M(*x*_*i *_- *x*_*j*_) ≥ 1 - *ξ*_*ijl*_

(2) *ξ*_*ijl *_≥ 0

(3) M ≥ 0

#### Other learning classifiers

Besides comparing learning classifiers LMNN and support vector machines with linear kernel (SVM_linear) and RBF kernel (SVM_rbf), we also applied several traditional classifiers including K-Nearest Neighbor Classifier (KNNC), Naïve Bayes Classifier (NBC), Nearest Mean Scaled Classifier (NMSC), Uncorrelated normal based quadratic Bayes Classifier recorded as UDC for the comparison study. The technical details about these learning classifiers can be found in reference [36].

### Data sets and experiments

The following three mass spectrometry data sets have been tested in our experiment:

1. High resolution time-of-flight (TOF) mass spectrometry (MS) proteomics data set from surface-enhanced laser/desorption ionization (SELDI) ProteinChip arrays on 121 ovarian cancer cases and 95 controls. The data sources can be accessed by FDA-NCI Clinical Proteomics at 

2. The breast cancer QC SELDI spectra data set was studied by Pusztai *et al*. [37]. Here, we utilized the data of 57 controls and 51 cases. The data set is available at: 

3. Matrix-assisted laser desorption/ionization time-of-flight (MALDI-TOF) liver disease data set was collected by Ressom *et al*. [38] for peak selection using ant colony optimization. The data set consists of 78 hepatocellular carcinoma (HCC, also called malignant hepatoma, a primary malignancy cancer of the liver), 51 cirrhosis (cirrhosis is a consequence of chronic liver disease characterized by replacement of liver tissue by fibrous scar tissue as well as regenerative nodules leading to progressive loss of liver function), and 72 normal. The spectra were binned with bin size of 100 ppm, and the dimension was reduced from 136,000 m/z values to 23846 m/z bins. Since the two liver diseases have similar symptoms but different treatments, our effort is focused on the classification of these two different diseases, or the identification of HCC and cirrhosis.

We process the data sets according to the methods described previously for peak detection and apply the SVMRFE and GLGS algorithms to the detected peak spectra data. The learning classifiers, listed in Table 1, are used for the training data and the testing data consisting of the feature sets chosen by SVMRFE and GLGS. In each experiment, 80% samples are randomly chosen for training, and the remaining 20% samples are tested. We ran the experiments 50 times for each combination of feature selection and learning classifiers, with the feature numbers from 5 to 100.

**Table 1:** Expected testing accuracy and standard errors (mean ± standard error, %) with classification models derived from best training, with the use of GLGS and SVMRFE feature selection algorithms and seven learning classifiers. Following the use of each feature selection algorithm on each data set, the best result as well as the classifier is highlighted in bold.

Learning classifier	GLGS			SVMRFE		
	
	Ovarian cancer	Breast cancer	Liver disease	Ovarian cancer	Breast cancer	Liver disease
KNNC	87.4 ± 5.8%	74.1 ± 6.9	80.9 ± 6.6	93.6 ± 3.8	82.8 ± 6.9	89.8 ± 3.9
NBC	78.9 ± 5.8	73.3 ± 8.5	87.1 ± 6.0	90.2 ± 4.5	74.1 ± 9.3	92.8 ± 4.1
NMSC	81.8 ± 5.2	76.2 ± 9.1	90.8 ± 4.9	92.2 ± 3.9	80.5 ± 8.0	94.3 ± 4.1
UDC	82.1 ± 5.6	76.9 ± 8.0	89.5 ± 5.9	91.8 ± 4.3	81.1 ± 7.4	90.4 ± 6.0
**SVM_linear**	89.6 ± 4.9	**85.6 ± 8.3**	95.8 ± 3.8	97.9 ± 2.0	89.9 ± 6.0	**98.2 ± 2.7**
**SVM_rbf**	**90.4 ± 4.3**	85.3 ± 7.9	**96.4 ± 3.3**	**98.2 ± 1.8**	**90.5 ± 6.1**	97.5 ± 3.1
LMNN	88.0 ± 4.9	75.5 ± 6.7	88.6 ± 4.7	97.4 ± 1.6	77.4 ± 5.8	91.6 ± 3.2

## Results

### Average testing under each dimension

**Figure 1:**
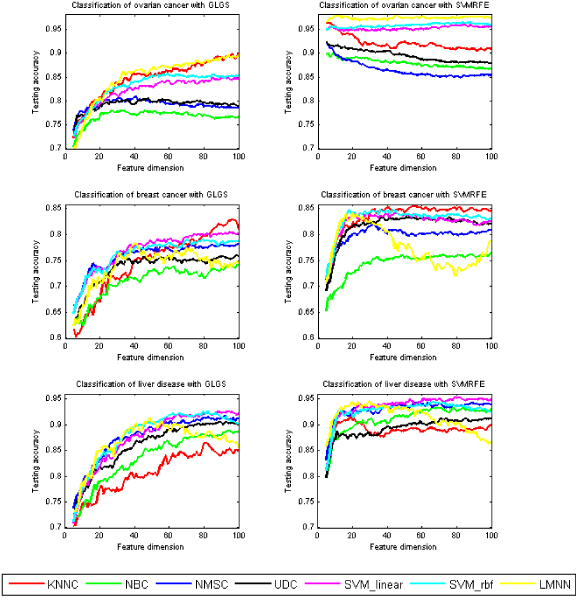
**Average testing accuracy after applying seven learning classifiers to the feature sets chosen by the GLGS (left) and the SVMRFE (right) algorithms on ovarian cancer (row 1), breast cancer (row 2), and liver disease (row 3) data sets, respectively**.

Figure 1 shows the average testing accuracy by using the seven classifiers for the feature sets chosen by GLGS and SVMRFE, with the feature numbers from 5 to 100. Regarding feature selection, SVMRFE is superior to GLGS in the testing of each type of MS data. In the testing for ovarian cancer data set, on average, LMNN is the best, followed by the SVM classifiers with linear kernel and rbf kernel. In the testing of the breast cancer data set, KNNC performs the best, followed by SVM classifiers with linear kernel and rbf kernel. In the testing of the liver disease data set, SVM classifiers outperformed other classifiers. Spanning over these three types of MS data, overall, SVM classifiers performed the best according to an evaluation of the testing accuracy and the stabilization. Worth mentioning is that, although LMNN has the best performance in testing the ovarian cancer data set, it did not fare well on the breast cancer and liver disease data sets, given the average from the feature dimension from 5 to 100. However, if we compare the testing accuracy of the feature sets with the number of features around 20 chosen by SVMRFE, LMNN delivered the most promising performance.

### Expected testing performance under best training

**Figure 2:**
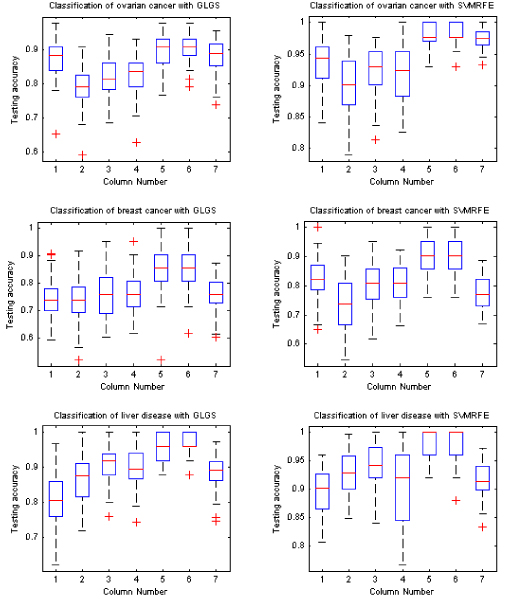
**Average testing accuracy with classification models derived from best training**. In each sub-figure, the results shown in column 1 to column 7 are obtained by using KNNC, NBC, NMSC, UDC, SVM_linear, SVM_rbf, and LMNN classifiers, respectively.

Besides comparing the average testing accuracy under each feature dimension from 5 to 100, we also compared the testing accuracy with the use of the classification models that are based on the best training. Figure 2 shows the box-plots of 50 expected testing accuracy values for each learning classifier with the feature selection methods of GLGS and SVMRFE, respectively. Table 1 lists the mean value and the standard error of the expected testing accuracy with the classification models derived from the best training. By comparing the box-plots on the left sub-figures and on the right sub-figures in Figure 1 and comparing the results shown in Table 1, we concluded that the SVMRFE outperformed GLGS and SVM classifiers showed remarkable advantages over other classifiers.

### Best testing performance under best training

**Figure 3:**
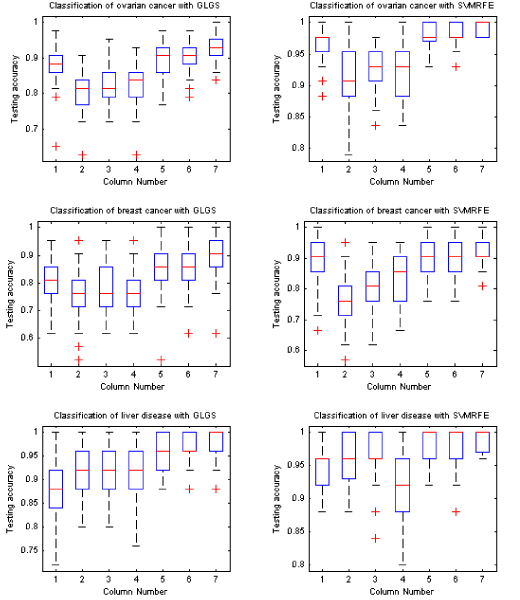
**Best testing accuracy with classification models derived from best training**. In each sub-figure, the results shown in column 1 to column 7 are obtained by using KNNC, NBC, NMSC, UDC, SVM_linear, SVM_rbf, and LMNN classifiers, respectively.

We also compared the best testing accuracy with the use of the classification models derived from the best training. Figure 3 shows the box-plots of 50 best testing accuracy values for each learning classifier with the feature selection methods of GLGS and SVMRFE, respectively. Table 2 lists the mean value and the standard error of the best testing accuracy with the classification models derived from the best training in each experiment. The results shown in Figure 3 and Table 2 demonstrated that SVMRFE is superior to GLGS, and that the LMNN delivered the best performance.

**Table 2:** Best testing accuracy and standard errors (mean ± standard error, %) with classification models derived from best training, with the use of GLGS and SVMRFE feature selection algorithms and seven learning classifiers. By using each feature selection algorithm on each data set, the best result as well as the classifier is highlighted in bold.

Learning classifier	GLGS			SVMRFE		
	
	Ovarian cancer	Breast cancer	Liver disease	Ovarian cancer	Breast cancer	Liver disease
KNNC	88.0 ± 5.8%	80.5 ± 8.6	88.3 ± 6.3	96.6 ± 2.9	87.9 ± 7.0	95.3 ± 3.4
NBC	79.9 ± 5.3	75.8 ± 9.0	90.8 ± 5.6	90.9 ± 4.5	76.0 ± 9.1	96.5 ± 3.7
NMSC	82.6 ± 5.1	77.8 ± 9.1	92.1 ± 4.4	92.6 ± 3.8	81.8 ± 7.6	96.5 ± 4.0
UDC	82.7 ± 5.4	78.0 ± 8.0	91.3 ± 5.6	92.5 ± 4.4	82.4 ± 7.7	91.7 ± 5.8
SVM_linear	89.6 ± 4.9	85.6 ± 8.3	95.8 ± 3.8	97.9 ± 2.0	89.9 ± 6.0	98.2 ± 2.7
SVM_rbf	90.4 ± 4.3	85.3 ± 7.9	96.4 ± 3.3	98.2 ± 1.8	90.5 ± 6.1	97.5 ± 3.1
**LMNN**	**93.1 ± 4.4**	**88.3 ± 7.4**	**97.4 ± 3.2**	**99.2 ± 1.1**	**91.7 ± 4.5**	**99.0 ± 1.8**

## Discussion

If we compare the results shown in Table 1 and Table 2, we found that the results obtained by using SVMs are the same in both tables, but the results of using other classifiers are different. In each experiment, with the use of other classifiers, there are multiple classification models, derived from the best trainings with different feature numbers. In this case, we calculated the average or expected testing value for Table 1 and obtained the best testing value for Table 2, respectively. On the other hand, by using SVM, we obtained a unique classification model derived from unique best training in each experiment; therefore, the results in Tables 1 and 2 are the same with the use of SVMs.

Regarding the expected testing performance under the best training, SVMs outperformed other classifiers. As for the best testing under best training, the best performance was associated with the learning classifier LMNN, which implies that distance metric learning is very promising for the classification of the MALDI-MS data., In these situations, it is the optimum classification model that delivers the best testing under the best training and, as such, is worthy of future investigation.

In comparison with the SVMRFE method, the GLGS feature selection method delivered a comparable and/or better performance in classifying microarray data; however, our experimental results showed that it does not perform as well as SVMRFE in classifying MALDI-MS data. This phenomenon is very interesting. In our opinion, it is caused by the difference between microarray data and MS data. Microarray data have a huge number of variables. It has a complicated correlation/interaction among genes as well as high redundancy. MALDI-MS data consist of mass/charge ratio values, after peak detection, correlation/interaction among peaks are generally not as complicated and much less redundancy exists. In such cases, SVMRFE is better than GLGS for classifying MS peak data.

## Competing interests

The authors declare that they have no competing interests.

## Authors' contributions

QL performed the study and drafted the manuscript; AHS initialized and supervised the study, provided supports, and finalized the draft; MQ worked on peak detection and helped with the manuscript drafting; ZC helped with the study and provided statistical analysis; JYY and MQY provided guidance; XH assisted the study and helped with manuscript editing; YD coordinated and assisted the project. All authors have read and approved the final manuscript.
